# Bis{4-[(1,3-benzodioxol-5-yl)meth­yl]piperazin-1-yl}methane

**DOI:** 10.1107/S1600536813028109

**Published:** 2013-10-19

**Authors:** Channappa N. Kavitha, Jerry P. Jasinski, Brian J. Anderson, H. S. Yathirajan, Manpreet Kaur

**Affiliations:** a Department of Studies in Chemistry, University of Mysore, Manasagangotri, Mysore 570 006, India; b Department of Chemistry, Keene State College, 229 Main Street, Keene, NH 03435-2001, USA

## Abstract

In the title compound, C_25_H_32_N_4_O_4_, both piperazine rings adopt a chair conformation. One of dioxolane ring systems is essentially planar [dihedral angle = 0.9 (2)°] while the other adopts a slightly disordered envelope conformation, the mean plane of the dioxolane ring being twisted by 3.6 (2)° from that of the benzene ring. The dihedral angle between the benzene rings is 69.9 (5)°. No classical hydrogen bonds were observed.

## Related literature

For the biological activity of piperazines, see: Choudhary *et al.* (2006 ▶); Kharb *et al.* (2012 ▶); Millan *et al.* (2001 ▶); Brockunier *et al.* (2004 ▶); Bogatcheva *et al.* (2006 ▶); Elliott (2011 ▶). For related structures, see: Capuano *et al.* (2000 ▶). For puckering parameters, see Cremer & Pople (1975 ▶).
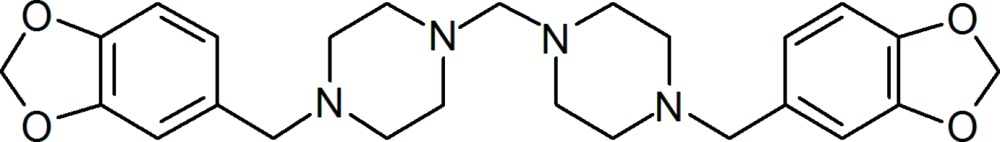


## Experimental

### 

#### Crystal data

C_25_H_32_N_4_O_4_
*M* *_r_* = 452.55
Orthorhombic, 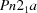

*a* = 38.8025 (10) Å
*b* = 9.7675 (2) Å
*c* = 6.09571 (13) Å
*V* = 2310.29 (10) Å^3^
*Z* = 4
Cu *K*α radiation
μ = 0.72 mm^−1^
*T* = 173 K
0.38 × 0.21 × 0.11 mm

#### Data collection

Agilent Xcalibur (Eos, Gemini) diffractometer
Absorption correction: multi-scan (*CrysAlis PRO* and *CrysAlis RED*; Agilent, 2012 ▶) *T*_min_ = 0.836, *T*_max_ = 1.000
13559 measured reflections
4199 independent reflections
3869 reflections with *I* > 2σ(*I*)
*R*_int_ = 0.046

#### Refinement

*R*[*F* ^2^ > 2σ(*F* ^2^)] = 0.041
*wR*(*F* ^2^) = 0.108
*S* = 1.02
4199 reflections
307 parameters
1 restraint
H atoms treated by a mixture of independent and constrained refinement
Δρ_max_ = 0.22 e Å^−3^
Δρ_min_ = −0.17 e Å^−3^
Absolute structure: Flack parameter determined using 1513 quotients [(I^+^)−(I^−^)]/[(I^+^)+(I^−^)] (Parsons & Flack, 2004 ▶)
Absolute structure parameter: −0.03 (15)

### 

Data collection: *CrysAlis PRO* (Agilent, 2012 ▶); cell refinement: *CrysAlis PRO*; data reduction: *CrysAlis RED* (Agilent, 2012 ▶); program(s) used to solve structure: *SUPERFLIP* (Palatinus & Chapuis, 2007 ▶); program(s) used to refine structure: *SHELXL2012* (Sheldrick, 2008 ▶); molecular graphics: *OLEX2* (Dolomanov *et al.*, 2009 ▶); software used to prepare material for publication: *OLEX2*.

## Supplementary Material

Crystal structure: contains datablock(s) I. DOI: 10.1107/S1600536813028109/fj2646sup1.cif

Structure factors: contains datablock(s) I. DOI: 10.1107/S1600536813028109/fj2646Isup2.hkl

Supplementary material file. DOI: 10.1107/S1600536813028109/fj2646Isup3.cml

Additional supplementary materials:  crystallographic information; 3D view; checkCIF report

## Figures and Tables

**Fig. 1.**
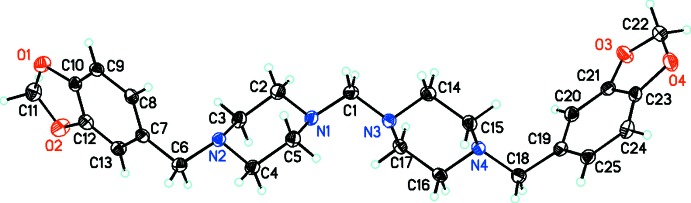
ORTEP drawing of (I) (C25H32N4O4) showing the labeling scheme with 30% probability displacement ellipsoids.

## supplementary crystallographic information

### 1. Comment

1-(3,4-Methylenedioxybenzyl)piperazine or 1-piperonylpiperazine is a psychoactive drug of the piperazine class and is used to synthesise the drug, piribedil, an antiparkinsonian agent (Millan *et al.*, 2001). The piperazine moiety is extensively employed to construct various bioactive molecules with anti-bacterial, antimalarial activity and as antipsychotic agents (Choudhary *et al.*, 2006). A valuable insight into recent advances on antimicrobial activity of piperazine derivatives is reported (Kharb *et al.*, 2012). Piperazines are among the most important building blocks in today's drug discovery and are found in biologically active compounds across a number of different therapeutic areas (Brockunier *et al.*, 2004; Bogatcheva *et al.*, 2006). A review on the current pharmacological and toxicological information for piperazine derivatives is described (Elliott, 2011). The crystal structure of N-piperonyl analogue of the atypical antipsychotic clozapine (Capuano *et al.*, 2000) is reported. In view of the above importance of piperonyl piperazines, this paper reports the crystal structure of the title compound, (I), C_25_H_32_N_4_O_4_, an unexpected product obtained during crystallization.

The title compound, (I), C_25_H_32_N_4_O_4_, crystallizes with one independent molecule in the asymmetric unit (Fig. 1). In the molecule, both piperazine rings adopt chair conformations (puckering parameters (N1/C2/C3/N2/C4/C5) Q, θ, and φ = 0.590 (3)Å, 0.0 (3)° and 173 (23)°; (N3/C14/C15/N4/C16/C17) Q, θ, and φ = 0.586 (3)Å, 0.7 (3)° and 182 (10)°,respectively (Cremer & Pople, 1975). One of the two five-membered dioxolane rings is planar while the other is in a slightly disordered envelope conformation (φ = 331.0 (18)°) where the mean plane of the dioxolane ring is twisted by 3.6 (1)° from that of the benzene ring. The dihedral angle between the mean planes of the two benzene rings is 69.9 (5)°. No classical hydrogen bonds were observed.

### 2. Experimental

0.5 g of 1-piperonylpiperazine (Sigma-Aldrich) was dissolved in 5 ml of methanol at 333 K with stirring for 10 min and left for slow evaporation. After two days, crystal formation was not observed. For the same compound, 5 ml of dimethyl formamide was added at 333 K with stirring for 15 min and left for slow evaporation. X-ray quality crystals were obtained and were used as such (m.p.: 308-312 K.)

### 3. Refinement

H1A and H1B were located by a difference map and refined isotropically. All of the remaining H atoms were placed in their calculated positions and then refined using the riding model with Atom—H lengths of 0.93Å (CH) or 0.97Å (CH_2_). Isotropic displacement parameters for these atoms were set to 1.2 (CH, CH_2_) times *U*_eq_ of the parent atom.

### Crystal data

**Table:**

C_25_H_32_N_4_O_4_	*D*_x_ = 1.312 Mg m^−^^3^
*M**_r_* = 452.55	Cu *K*α radiation, λ = 1.54184 Å
Orthorhombic, *P**n*2_1_*a*	Cell parameters from 5154 reflections
*a* = 38.8025 (10) Å	θ = 4.5–72.4°
*b* = 9.7675 (2) Å	µ = 0.72 mm^−^^1^
*c* = 6.09571 (13) Å	*T* = 173 K
*V* = 2310.29 (10) Å^3^	Irregular, colourless
*Z* = 4	0.38 × 0.21 × 0.11 mm
*F*(000) = 968	

### Data collection

**Table:**

Agilent Xcalibur (Eos, Gemini) diffractometer	4199 independent reflections
Radiation source: Enhance (Cu) X-ray Source	3869 reflections with *I* > 2σ(*I*)
Detector resolution: 16.0416 pixels mm^-1^	*R*_int_ = 0.046
ω scans	θ_max_ = 72.6°, θ_min_ = 4.6°
Absorption correction: multi-scan (*CrysAlis PRO* and *CrysAlis RED*; Agilent, 2012)	*h* = −43→48
*T*_min_ = 0.836, *T*_max_ = 1.000	*k* = −11→12
13559 measured reflections	*l* = −5→7

### Refinement

**Table:**

Refinement on *F*^2^	H atoms treated by a mixture of independent and constrained refinement
Least-squares matrix: full	*w* = 1/[σ^2^(*F*_o_^2^) + (0.0485*P*)^2^ + 0.6698*P*] where *P* = (*F*_o_^2^ + 2*F*_c_^2^)/3
*R*[*F*^2^ > 2σ(*F*^2^)] = 0.041	(Δ/σ)_max_ < 0.001
*wR*(*F*^2^) = 0.108	Δρ_max_ = 0.22 e Å^−^^3^
*S* = 1.02	Δρ_min_ = −0.17 e Å^−^^3^
4199 reflections	Extinction correction: *SHELXL2012* (Sheldrick, 2008), Fc^*^=kFc[1+0.001xFc^2^λ^3^/sin(2θ)]^-1/4^
307 parameters	Extinction coefficient: 0.0016 (2)
1 restraint	Absolute structure: Flack parameter determined using 1513 quotients [(I+)-(I-)]/[(I+)+(I-)] (Parsons & Flack, 2004)
Hydrogen site location: mixed	Absolute structure parameter: −0.03 (15)

### Special details

**Table:**

Geometry. All esds (except the esd in the dihedral angle between two l.s. planes) are estimated using the full covariance matrix. The cell esds are taken into account individually in the estimation of esds in distances, angles and torsion angles; correlations between esds in cell parameters are only used when they are defined by crystal symmetry. An approximate (isotropic) treatment of cell esds is used for estimating esds involving l.s. planes.

### Fractional atomic coordinates and isotropic or equivalent isotropic displacement parameters (Å^2^)

**Table:**

	*x*	*y*	*z*	*U*_iso_*/*U*_eq_	
O1	0.31516 (6)	0.5691 (3)	1.0003 (4)	0.0425 (5)	
O2	0.31379 (7)	0.7799 (3)	0.8258 (4)	0.0534 (7)	
O3	0.75845 (6)	0.5660 (3)	0.1266 (4)	0.0452 (6)	
O4	0.77389 (6)	0.4101 (3)	−0.1424 (4)	0.0431 (6)	
N1	0.50857 (6)	0.5885 (3)	0.2715 (4)	0.0316 (6)	
N2	0.43489 (6)	0.5829 (3)	0.2856 (4)	0.0283 (5)	
N3	0.56439 (6)	0.5613 (3)	0.1084 (4)	0.0305 (5)	
N4	0.62717 (6)	0.6657 (3)	−0.0775 (4)	0.0287 (5)	
C0AA	0.54264 (8)	0.5299 (4)	0.2949 (5)	0.0359 (7)	
C2	0.48991 (8)	0.5896 (4)	0.4788 (5)	0.0388 (8)	
H2A	0.5031	0.6394	0.5879	0.047*	
H2B	0.4868	0.4965	0.5306	0.047*	
C3	0.45513 (8)	0.6567 (4)	0.4487 (5)	0.0363 (7)	
H3A	0.4429	0.6576	0.5875	0.044*	
H3B	0.4583	0.7508	0.4018	0.044*	
C4	0.45353 (7)	0.5831 (4)	0.0781 (4)	0.0320 (6)	
H4A	0.4566	0.6766	0.0279	0.038*	
H4B	0.4403	0.5341	−0.0317	0.038*	
C5	0.48843 (8)	0.5158 (3)	0.1056 (5)	0.0335 (7)	
H5A	0.4854	0.4211	0.1496	0.040*	
H5B	0.5007	0.5169	−0.0332	0.040*	
C6	0.40051 (8)	0.6420 (3)	0.2635 (5)	0.0336 (7)	
H6A	0.3892	0.6021	0.1368	0.040*	
H6B	0.4026	0.7397	0.2382	0.040*	
C7	0.37846 (7)	0.6181 (3)	0.4645 (5)	0.0290 (6)	
C8	0.37856 (7)	0.4919 (3)	0.5700 (5)	0.0291 (6)	
H8	0.3930	0.4234	0.5177	0.035*	
C9	0.35775 (7)	0.4641 (3)	0.7517 (5)	0.0304 (6)	
H9	0.3578	0.3789	0.8197	0.036*	
C10	0.33726 (7)	0.5687 (3)	0.8245 (4)	0.0297 (6)	
C11	0.30024 (9)	0.7027 (4)	1.0040 (6)	0.0445 (8)	
H11A	0.2754	0.6961	0.9910	0.053*	
H11B	0.3056	0.7478	1.1416	0.053*	
C12	0.33670 (8)	0.6945 (3)	0.7208 (5)	0.0331 (7)	
C13	0.35674 (8)	0.7227 (3)	0.5419 (5)	0.0337 (7)	
H13	0.3560	0.8079	0.4740	0.040*	
C14	0.59693 (8)	0.4870 (3)	0.1253 (5)	0.0342 (7)	
H14A	0.5924	0.3894	0.1294	0.041*	
H14B	0.6085	0.5122	0.2605	0.041*	
C15	0.62001 (8)	0.5196 (3)	−0.0679 (5)	0.0332 (7)	
H15A	0.6415	0.4695	−0.0540	0.040*	
H15B	0.6089	0.4909	−0.2027	0.040*	
C16	0.59437 (7)	0.7386 (3)	−0.0990 (5)	0.0311 (6)	
H16A	0.5830	0.7110	−0.2337	0.037*	
H16B	0.5986	0.8364	−0.1065	0.037*	
C17	0.57131 (7)	0.7078 (3)	0.0934 (5)	0.0320 (6)	
H17A	0.5823	0.7388	0.2275	0.038*	
H17B	0.5498	0.7570	0.0770	0.038*	
C18	0.65041 (7)	0.7011 (3)	−0.2577 (5)	0.0341 (7)	
H18A	0.6544	0.7991	−0.2567	0.041*	
H18B	0.6395	0.6779	−0.3959	0.041*	
C19	0.68447 (7)	0.6275 (3)	−0.2412 (5)	0.0310 (6)	
C20	0.70492 (8)	0.6439 (4)	−0.0521 (5)	0.0333 (7)	
H20	0.6987	0.7043	0.0590	0.040*	
C21	0.73433 (7)	0.5672 (4)	−0.0390 (4)	0.0314 (6)	
C22	0.78104 (9)	0.4551 (4)	0.0760 (6)	0.0439 (8)	
H22A	0.7775	0.3806	0.1787	0.053*	
H22B	0.8048	0.4848	0.0872	0.053*	
C23	0.74391 (7)	0.4755 (3)	−0.2007 (5)	0.0323 (7)	
C24	0.72513 (8)	0.4605 (4)	−0.3887 (5)	0.0367 (7)	
H24	0.7320	0.4014	−0.4999	0.044*	
C25	0.69508 (8)	0.5383 (3)	−0.4056 (5)	0.0325 (7)	
H25	0.6817	0.5302	−0.5314	0.039*	
H0AA	0.5513 (8)	0.564 (4)	0.426 (5)	0.031 (9)*	
H0AB	0.5410 (9)	0.429 (4)	0.313 (6)	0.040 (10)*	

### Atomic displacement parameters (Å^2^)

**Table:**

	*U*^11^	*U*^22^	*U*^33^	*U*^12^	*U*^13^	*U*^23^
O1	0.0476 (13)	0.0446 (14)	0.0352 (11)	0.0079 (11)	0.0122 (10)	0.0003 (10)
O2	0.0654 (17)	0.0392 (15)	0.0557 (16)	0.0191 (12)	0.0233 (13)	0.0014 (12)
O3	0.0382 (12)	0.0574 (16)	0.0399 (12)	0.0129 (12)	−0.0079 (9)	−0.0052 (12)
O4	0.0318 (12)	0.0503 (14)	0.0471 (14)	0.0094 (10)	0.0022 (10)	−0.0030 (11)
N1	0.0285 (12)	0.0389 (15)	0.0275 (12)	−0.0058 (11)	−0.0001 (9)	0.0010 (11)
N2	0.0286 (12)	0.0321 (13)	0.0240 (11)	−0.0032 (10)	0.0007 (9)	−0.0009 (10)
N3	0.0267 (12)	0.0288 (13)	0.0361 (13)	−0.0029 (10)	−0.0018 (9)	0.0050 (11)
N4	0.0266 (12)	0.0259 (13)	0.0336 (13)	−0.0005 (10)	−0.0013 (9)	0.0018 (10)
C0AA	0.0338 (16)	0.0421 (19)	0.0318 (15)	−0.0032 (14)	−0.0035 (12)	0.0075 (14)
C2	0.0345 (15)	0.055 (2)	0.0270 (14)	−0.0094 (15)	−0.0037 (12)	−0.0020 (15)
C3	0.0354 (16)	0.0444 (19)	0.0290 (15)	−0.0069 (14)	0.0037 (12)	−0.0073 (13)
C4	0.0331 (15)	0.0372 (16)	0.0258 (13)	−0.0052 (13)	−0.0004 (11)	−0.0007 (13)
C5	0.0332 (15)	0.0394 (18)	0.0280 (15)	−0.0034 (13)	0.0005 (12)	−0.0041 (13)
C6	0.0344 (16)	0.0326 (16)	0.0338 (15)	0.0020 (13)	0.0008 (12)	0.0037 (13)
C7	0.0280 (13)	0.0308 (16)	0.0282 (14)	0.0000 (12)	−0.0032 (11)	−0.0020 (11)
C8	0.0259 (14)	0.0281 (16)	0.0333 (15)	0.0029 (11)	−0.0011 (11)	−0.0044 (11)
C9	0.0304 (15)	0.0276 (14)	0.0333 (16)	−0.0002 (12)	−0.0040 (12)	0.0019 (12)
C10	0.0262 (13)	0.0356 (16)	0.0273 (13)	−0.0020 (12)	−0.0006 (11)	−0.0027 (12)
C11	0.0441 (18)	0.044 (2)	0.0451 (19)	0.0063 (16)	0.0116 (15)	−0.0095 (16)
C12	0.0326 (15)	0.0330 (16)	0.0337 (15)	0.0053 (13)	−0.0020 (12)	−0.0068 (12)
C13	0.0359 (15)	0.0263 (15)	0.0387 (16)	0.0031 (13)	−0.0018 (13)	0.0010 (13)
C14	0.0326 (16)	0.0265 (15)	0.0435 (17)	−0.0010 (12)	−0.0020 (12)	0.0064 (13)
C15	0.0298 (15)	0.0261 (15)	0.0437 (17)	0.0030 (12)	0.0007 (13)	0.0031 (13)
C16	0.0299 (15)	0.0245 (15)	0.0389 (16)	0.0005 (11)	−0.0055 (12)	0.0038 (12)
C17	0.0269 (14)	0.0290 (16)	0.0401 (16)	0.0022 (12)	−0.0029 (12)	−0.0008 (13)
C18	0.0313 (15)	0.0340 (17)	0.0369 (16)	−0.0019 (13)	−0.0020 (12)	0.0048 (13)
C19	0.0292 (14)	0.0316 (15)	0.0323 (15)	−0.0042 (12)	0.0028 (11)	0.0048 (12)
C20	0.0321 (15)	0.0352 (17)	0.0325 (15)	0.0010 (13)	0.0024 (12)	−0.0015 (13)
C21	0.0313 (14)	0.0359 (16)	0.0269 (14)	−0.0035 (13)	0.0009 (11)	0.0040 (13)
C22	0.0359 (17)	0.054 (2)	0.0422 (19)	0.0114 (16)	0.0030 (14)	0.0095 (16)
C23	0.0260 (14)	0.0335 (17)	0.0374 (16)	−0.0005 (12)	0.0064 (12)	0.0051 (13)
C24	0.0326 (16)	0.0390 (17)	0.0385 (17)	−0.0052 (13)	0.0093 (13)	−0.0031 (14)
C25	0.0305 (15)	0.0373 (17)	0.0298 (15)	−0.0070 (12)	−0.0001 (11)	0.0036 (12)

### Geometric parameters (Å, º)

**Table:**

O1—C10	1.373 (3)	C7—C8	1.390 (4)
O1—C11	1.428 (4)	C7—C13	1.406 (4)
O2—C11	1.422 (4)	C8—H8	0.9300
O2—C12	1.377 (4)	C8—C9	1.397 (4)
O3—C21	1.377 (3)	C9—H9	0.9300
O3—C22	1.427 (4)	C9—C10	1.369 (4)
O4—C22	1.429 (4)	C10—C12	1.382 (5)
O4—C23	1.374 (4)	C11—H11A	0.9700
N1—C0AA	1.447 (4)	C11—H11B	0.9700
N1—C2	1.456 (4)	C12—C13	1.367 (4)
N1—C5	1.462 (4)	C13—H13	0.9300
N2—C3	1.458 (4)	C14—H14A	0.9700
N2—C4	1.457 (3)	C14—H14B	0.9700
N2—C6	1.460 (4)	C14—C15	1.513 (4)
N3—C0AA	1.449 (4)	C15—H15A	0.9700
N3—C14	1.460 (4)	C15—H15B	0.9700
N3—C17	1.459 (4)	C16—H16A	0.9700
N4—C15	1.455 (4)	C16—H16B	0.9700
N4—C16	1.464 (4)	C16—C17	1.505 (4)
N4—C18	1.462 (4)	C17—H17A	0.9700
C0AA—H0AA	0.93 (3)	C17—H17B	0.9700
C0AA—H0AB	0.99 (4)	C18—H18A	0.9700
C2—H2A	0.9700	C18—H18B	0.9700
C2—H2B	0.9700	C18—C19	1.508 (4)
C2—C3	1.512 (4)	C19—C20	1.409 (4)
C3—H3A	0.9700	C19—C25	1.390 (4)
C3—H3B	0.9700	C20—H20	0.9300
C4—H4A	0.9700	C20—C21	1.367 (5)
C4—H4B	0.9700	C21—C23	1.382 (4)
C4—C5	1.515 (4)	C22—H22A	0.9700
C5—H5A	0.9700	C22—H22B	0.9700
C5—H5B	0.9700	C23—C24	1.366 (5)
C6—H6A	0.9700	C24—H24	0.9300
C6—H6B	0.9700	C24—C25	1.396 (5)
C6—C7	1.512 (4)	C25—H25	0.9300
			
C10—O1—C11	105.5 (3)	O1—C11—H11B	109.9
C12—O2—C11	105.8 (3)	O2—C11—O1	108.8 (2)
C21—O3—C22	105.4 (3)	O2—C11—H11A	109.9
C23—O4—C22	105.2 (2)	O2—C11—H11B	109.9
C0AA—N1—C2	111.8 (2)	H11A—C11—H11B	108.3
C0AA—N1—C5	111.4 (3)	O2—C12—C10	109.7 (3)
C2—N1—C5	109.8 (2)	C13—C12—O2	128.0 (3)
C3—N2—C6	111.1 (2)	C13—C12—C10	122.4 (3)
C4—N2—C3	108.9 (2)	C7—C13—H13	121.2
C4—N2—C6	111.9 (2)	C12—C13—C7	117.5 (3)
C0AA—N3—C14	110.1 (2)	C12—C13—H13	121.2
C0AA—N3—C17	111.3 (3)	N3—C14—H14A	109.5
C14—N3—C17	109.4 (2)	N3—C14—H14B	109.5
C15—N4—C16	108.3 (2)	N3—C14—C15	110.6 (3)
C15—N4—C18	112.3 (3)	H14A—C14—H14B	108.1
C16—N4—C18	110.7 (2)	C15—C14—H14A	109.5
N1—C0AA—N3	111.8 (3)	C15—C14—H14B	109.5
N1—C0AA—H0AA	106 (2)	N4—C15—C14	110.5 (3)
N1—C0AA—H0AB	110 (2)	N4—C15—H15A	109.5
N3—C0AA—H0AA	113 (2)	N4—C15—H15B	109.5
N3—C0AA—H0AB	109 (2)	C14—C15—H15A	109.5
H0AA—C0AA—H0AB	106 (3)	C14—C15—H15B	109.5
N1—C2—H2A	109.7	H15A—C15—H15B	108.1
N1—C2—H2B	109.7	N4—C16—H16A	109.6
N1—C2—C3	110.0 (3)	N4—C16—H16B	109.6
H2A—C2—H2B	108.2	N4—C16—C17	110.5 (2)
C3—C2—H2A	109.7	H16A—C16—H16B	108.1
C3—C2—H2B	109.7	C17—C16—H16A	109.6
N2—C3—C2	110.4 (3)	C17—C16—H16B	109.6
N2—C3—H3A	109.6	N3—C17—C16	110.7 (2)
N2—C3—H3B	109.6	N3—C17—H17A	109.5
C2—C3—H3A	109.6	N3—C17—H17B	109.5
C2—C3—H3B	109.6	C16—C17—H17A	109.5
H3A—C3—H3B	108.1	C16—C17—H17B	109.5
N2—C4—H4A	109.6	H17A—C17—H17B	108.1
N2—C4—H4B	109.6	N4—C18—H18A	109.2
N2—C4—C5	110.3 (2)	N4—C18—H18B	109.2
H4A—C4—H4B	108.1	N4—C18—C19	112.2 (2)
C5—C4—H4A	109.6	H18A—C18—H18B	107.9
C5—C4—H4B	109.6	C19—C18—H18A	109.2
N1—C5—C4	110.1 (3)	C19—C18—H18B	109.2
N1—C5—H5A	109.6	C20—C19—C18	119.6 (3)
N1—C5—H5B	109.6	C25—C19—C18	120.7 (3)
C4—C5—H5A	109.6	C25—C19—C20	119.6 (3)
C4—C5—H5B	109.6	C19—C20—H20	121.5
H5A—C5—H5B	108.2	C21—C20—C19	117.1 (3)
N2—C6—H6A	109.1	C21—C20—H20	121.5
N2—C6—H6B	109.1	O3—C21—C23	109.5 (3)
N2—C6—C7	112.4 (2)	C20—C21—O3	127.9 (3)
H6A—C6—H6B	107.9	C20—C21—C23	122.6 (3)
C7—C6—H6A	109.1	O3—C22—O4	108.4 (3)
C7—C6—H6B	109.1	O3—C22—H22A	110.0
C8—C7—C6	120.7 (3)	O3—C22—H22B	110.0
C8—C7—C13	119.4 (3)	O4—C22—H22A	110.0
C13—C7—C6	119.9 (3)	O4—C22—H22B	110.0
C7—C8—H8	118.7	H22A—C22—H22B	108.4
C7—C8—C9	122.5 (3)	O4—C23—C21	110.2 (3)
C9—C8—H8	118.7	C24—C23—O4	128.2 (3)
C8—C9—H9	121.7	C24—C23—C21	121.6 (3)
C10—C9—C8	116.6 (3)	C23—C24—H24	121.6
C10—C9—H9	121.7	C23—C24—C25	116.7 (3)
O1—C10—C12	110.2 (3)	C25—C24—H24	121.6
C9—C10—O1	128.2 (3)	C19—C25—C24	122.4 (3)
C9—C10—C12	121.6 (3)	C19—C25—H25	118.8
O1—C11—H11A	109.9	C24—C25—H25	118.8
			
O1—C10—C12—O2	−0.7 (4)	C9—C10—C12—C13	−0.8 (5)
O1—C10—C12—C13	179.1 (3)	C10—O1—C11—O2	0.5 (3)
O2—C12—C13—C7	179.6 (3)	C10—C12—C13—C7	−0.1 (4)
O3—C21—C23—O4	1.5 (4)	C11—O1—C10—C9	179.9 (3)
O3—C21—C23—C24	−177.3 (3)	C11—O1—C10—C12	0.1 (3)
O4—C23—C24—C25	179.0 (3)	C11—O2—C12—C10	1.0 (4)
N1—C2—C3—N2	59.5 (4)	C11—O2—C12—C13	−178.8 (3)
N2—C4—C5—N1	−59.0 (3)	C12—O2—C11—O1	−0.9 (4)
N2—C6—C7—C8	−42.1 (4)	C13—C7—C8—C9	0.1 (4)
N2—C6—C7—C13	140.0 (3)	C14—N3—C0AA—N1	−173.1 (3)
N3—C14—C15—N4	59.2 (3)	C14—N3—C17—C16	57.2 (3)
N4—C16—C17—N3	−59.4 (3)	C15—N4—C16—C17	59.5 (3)
N4—C18—C19—C20	−58.0 (4)	C15—N4—C18—C19	−58.6 (3)
N4—C18—C19—C25	118.7 (3)	C16—N4—C15—C14	−59.4 (3)
C0AA—N1—C2—C3	177.7 (3)	C16—N4—C18—C19	−179.8 (3)
C0AA—N1—C5—C4	−177.6 (3)	C17—N3—C0AA—N1	65.3 (3)
C0AA—N3—C14—C15	−179.6 (3)	C17—N3—C14—C15	−56.9 (3)
C0AA—N3—C17—C16	179.1 (2)	C18—N4—C15—C14	178.0 (2)
C2—N1—C0AA—N3	−164.6 (3)	C18—N4—C16—C17	−176.9 (2)
C2—N1—C5—C4	58.0 (3)	C18—C19—C20—C21	175.4 (3)
C3—N2—C4—C5	59.2 (3)	C18—C19—C25—C24	−175.0 (3)
C3—N2—C6—C7	−69.1 (3)	C19—C20—C21—O3	179.3 (3)
C4—N2—C3—C2	−59.5 (3)	C19—C20—C21—C23	−0.8 (5)
C4—N2—C6—C7	169.0 (3)	C20—C19—C25—C24	1.7 (5)
C5—N1—C0AA—N3	72.2 (3)	C20—C21—C23—O4	−178.4 (3)
C5—N1—C2—C3	−58.2 (4)	C20—C21—C23—C24	2.9 (5)
C6—N2—C3—C2	176.9 (2)	C21—O3—C22—O4	11.6 (4)
C6—N2—C4—C5	−177.7 (3)	C21—C23—C24—C25	−2.5 (5)
C6—C7—C8—C9	−177.8 (3)	C22—O3—C21—C20	171.8 (3)
C6—C7—C13—C12	178.3 (3)	C22—O3—C21—C23	−8.1 (3)
C7—C8—C9—C10	−0.9 (4)	C22—O4—C23—C21	5.7 (4)
C8—C7—C13—C12	0.4 (4)	C22—O4—C23—C24	−175.6 (3)
C8—C9—C10—O1	−178.6 (3)	C23—O4—C22—O3	−10.7 (4)
C8—C9—C10—C12	1.2 (4)	C23—C24—C25—C19	0.2 (5)
C9—C10—C12—O2	179.4 (3)	C25—C19—C20—C21	−1.4 (4)
